# Supplementary material for: Hot spot prediction in protein-protein interactions by an ensemble system
Source: BMC Syst Biol. 2018 Dec 31;12(Suppl 9):132. doi: 10.1186/s12918-018-0665-8 (PMC6311905; doi:10.1186/s12918-018-0665-8)
Supplement: Supplementary file 2 — All relevant MATLAB code. A simple Matlab implement of our predictor. (ZIP 19,742 kb) [file 12918_2018_665_MOESM2_ESM.zip › Additional file 2/Read ASA/ASA website/dbMPIKT/4Y61A.html]

 NetSurfP - Protein Surface Accessibility and Secondary Structure Predictions

  

|  |  |  |
| --- | --- | --- |
|  |  | NetSurfP - Protein Surface Accessibility and Secondary Structure Predictions  Technical University of Denmark |

  


---

```
# For publication of results, please cite:
# A generic method for assignment of reliability scores applied to solvent accessibility predictions.
# Bent Petersen, Thomas Nordahl Petersen, Pernille Andersen, Morten Nielsen and Claus Lundegaard
# BMC Structural Biology 2009, 9:51 doi:10.1186/1472-6807-9-51
#
# Column 1: Class assignment - B for buried or E for Exposed - Threshold: 25% exposure, but not based on RSA
# Column 2: Amino acid
# Column 3: Sequence name
# Column 4: Amino acid number
# Column 5: Relative Surface Accessibility - RSA
# Column 6: Absolute Surface Accessibility
# Column 7: Z-fit score for RSA prediction
# Column 8: Probability for Alpha-Helix
# Column 9: Probability for Beta-strand
# Column 10: Probability for Coil
E E  4Y61A                  1    0.895 156.269   0.973   0.003   0.003   0.994
E T  4Y61A                  2    0.514  71.333   0.091   0.005   0.045   0.951
E P  4Y61A                  3    0.390  55.270   0.488   0.018   0.047   0.935
B P  4Y61A                  4    0.115  16.304  -0.854   0.020   0.205   0.775
E R  4Y61A                  5    0.514 117.592   1.324   0.003   0.718   0.279
B F  4Y61A                  6    0.129  25.850   0.249   0.002   0.816   0.182
E T  4Y61A                  7    0.332  46.007   0.396   0.018   0.846   0.136
E R  4Y61A                  8    0.463 106.073   0.505   0.004   0.616   0.381
E T  4Y61A                  9    0.475  65.938   1.093   0.005   0.262   0.733
E P  4Y61A                 10    0.288  40.938  -0.831   0.005   0.045   0.951
E V  4Y61A                 11    0.627  96.401   0.480   0.004   0.197   0.799
E D  4Y61A                 12    0.628  90.509   0.174   0.004   0.420   0.576
B Q  4Y61A                 13    0.192  34.273   1.393   0.002   0.816   0.182
E T  4Y61A                 14    0.527  73.109   1.070   0.001   0.900   0.099
B G  4Y61A                 15    0.076   5.997   0.390   0.001   0.900   0.099
E V  4Y61A                 16    0.459  70.502   1.016   0.003   0.718   0.279
E S  4Y61A                 17    0.457  53.514   0.371   0.004   0.197   0.799
E G  4Y61A                 18    0.729  57.388  -0.803   0.005   0.015   0.979
E G  4Y61A                 19    0.399  31.425   1.070   0.004   0.085   0.910
E V  4Y61A                 20    0.533  81.845   1.356   0.004   0.616   0.381
B A  4Y61A                 21    0.044   4.838   0.712   0.001   0.959   0.040
E S  4Y61A                 22    0.368  43.176   1.970   0.001   0.959   0.040
B F  4Y61A                 23    0.038   7.546   0.629   0.001   0.959   0.040
E I  4Y61A                 24    0.338  62.474   1.713   0.001   0.959   0.040
B C  4Y61A                 25    0.017   2.387   1.272   0.001   0.959   0.040
E Q  4Y61A                 26    0.393  70.226   1.736   0.001   0.959   0.040
B A  4Y61A                 27    0.039   4.331  -0.905   0.001   0.959   0.040
E T  4Y61A                 28    0.478  66.312   0.655   0.002   0.816   0.182
E G  4Y61A                 29    0.413  32.511  -1.578   0.005   0.336   0.660
E D  4Y61A                 30    0.442  63.649   0.085   0.004   0.138   0.858
E P  4Y61A                 31    0.321  45.592  -0.855   0.004   0.138   0.858
E R  4Y61A                 32    0.548 125.469   0.727   0.004   0.138   0.858
B P  4Y61A                 33    0.083  11.749  -0.506   0.004   0.514   0.481
E K  4Y61A                 34    0.360  73.990   1.277   0.001   0.900   0.099
B I  4Y61A                 35    0.039   7.160   0.058   0.001   0.959   0.040
E V  4Y61A                 36    0.255  39.240   1.906   0.001   0.959   0.040
B W  4Y61A                 37    0.033   7.985   0.281   0.006   0.962   0.032
B N  4Y61A                 38    0.235  34.477   1.162   0.001   0.959   0.040
B K  4Y61A                 39    0.223  45.851   0.993   0.002   0.816   0.182
E K  4Y61A                 40    0.595 122.289  -0.102   0.005   0.015   0.979
E G  4Y61A                 41    0.731  57.506  -1.202   0.005   0.015   0.979
E K  4Y61A                 42    0.503 103.385   0.654   0.002   0.816   0.182
E K  4Y61A                 43    0.498 102.541   1.025   0.001   0.900   0.099
B V  4Y61A                 44    0.069  10.636  -0.065   0.023   0.655   0.322
E S  4Y61A                 45    0.472  55.365   0.618   0.004   0.420   0.576
E N  4Y61A                 46    0.547  80.066  -0.591   0.004   0.197   0.799
E Q  4Y61A                 47    0.566 101.105  -1.938   0.005   0.015   0.979
E R  4Y61A                 48    0.644 147.384  -1.652   0.005   0.045   0.951
E F  4Y61A                 49    0.444  89.071  -1.035   0.004   0.420   0.576
E E  4Y61A                 50    0.293  51.187   0.069   0.002   0.816   0.182
E V  4Y61A                 51    0.269  41.422   0.110   0.001   0.959   0.040
B I  4Y61A                 52    0.144  26.696   0.126   0.001   0.959   0.040
E E  4Y61A                 53    0.283  49.388  -0.463   0.001   0.959   0.040
B F  4Y61A                 54    0.219  43.973  -0.428   0.001   0.900   0.099
E D  4Y61A                 55    0.446  64.341  -0.917   0.004   0.616   0.381
E D  4Y61A                 56    0.606  87.310  -1.642   0.004   0.138   0.858
E G  4Y61A                 57    0.570  44.851  -2.351   0.005   0.015   0.979
E S  4Y61A                 58    0.536  62.854  -0.859   0.004   0.138   0.858
B G  4Y61A                 59    0.244  19.187  -0.193   0.004   0.616   0.381
B S  4Y61A                 60    0.083   9.751  -0.058   0.002   0.816   0.182
B V  4Y61A                 61    0.107  16.369   0.065   0.001   0.900   0.099
B L  4Y61A                 62    0.025   4.541   0.485   0.001   0.959   0.040
B R  4Y61A                 63    0.181  41.564   0.888   0.001   0.959   0.040
B I  4Y61A                 64    0.031   5.698  -0.034   0.001   0.900   0.099
E Q  4Y61A                 65    0.346  61.831   0.985   0.003   0.718   0.279
E P  4Y61A                 66    0.498  70.595  -0.277   0.004   0.616   0.381
B L  4Y61A                 67    0.170  31.035  -0.326   0.005   0.336   0.660
E R  4Y61A                 68    0.509 116.584  -0.298   0.005   0.262   0.733
E T  4Y61A                 69    0.462  64.121  -0.115   0.005   0.262   0.733
E P  4Y61A                 70    0.509  72.298  -0.642   0.004   0.138   0.858
E R  4Y61A                 71    0.628 143.858  -0.567   0.004   0.085   0.910
B D  4Y61A                 72    0.172  24.828  -1.195   0.004   0.138   0.858
E E  4Y61A                 73    0.474  82.808  -0.149   0.004   0.138   0.858
B A  4Y61A                 74    0.089   9.786  -1.392   0.003   0.718   0.279
E I  4Y61A                 75    0.358  66.156   2.070   0.001   0.959   0.040
B Y  4Y61A                 76    0.037   7.843   0.447   0.001   0.959   0.040
B E  4Y61A                 77    0.206  35.918   1.991   0.001   0.959   0.040
B C  4Y61A                 78    0.012   1.713   1.704   0.001   0.959   0.040
E V  4Y61A                 79    0.246  37.795   2.077   0.001   0.959   0.040
B A  4Y61A                 80    0.022   2.424   0.614   0.001   0.959   0.040
E S  4Y61A                 81    0.369  43.270   0.764   0.001   0.959   0.040
B N  4Y61A                 82    0.127  18.622  -0.695   0.004   0.514   0.481
E N  4Y61A                 83    0.638  93.403  -0.097   0.005   0.045   0.951
E V  4Y61A                 84    0.520  79.847  -1.178   0.004   0.085   0.910
E G  4Y61A                 85    0.477  37.579  -0.917   0.005   0.336   0.660
E E  4Y61A                 86    0.512  89.359  -0.232   0.004   0.616   0.381
E I  4Y61A                 87    0.393  72.779   0.224   0.001   0.900   0.099
E S  4Y61A                 88    0.404  47.314  -0.050   0.001   0.900   0.099
B V  4Y61A                 89    0.172  26.436   0.823   0.001   0.959   0.040
E S  4Y61A                 90    0.404  47.407   0.894   0.001   0.959   0.040
B T  4Y61A                 91    0.036   4.938  -0.157   0.001   0.959   0.040
E R  4Y61A                 92    0.283  64.715   1.335   0.001   0.959   0.040
B L  4Y61A                 93    0.023   4.303   0.915   0.001   0.959   0.040
E T  4Y61A                 94    0.234  32.511   1.627   0.001   0.959   0.040
B V  4Y61A                 95    0.029   4.488   0.567   0.001   0.959   0.040
E L  4Y61A                 96    0.278  50.993   1.075   0.001   0.900   0.099
B R  4Y61A                 97    0.207  47.472   0.451   0.004   0.616   0.381
E E  4Y61A                 98    0.302  52.724   0.295   0.020   0.205   0.775
E D  4Y61A                 99    0.460  66.243  -0.954   0.018   0.088   0.893
E Q  4Y61A                100    0.624 111.446  -0.864   0.018   0.088   0.893
B I  4Y61A                101    0.261  48.322  -1.056   0.018   0.088   0.893
E P  4Y61A                102    0.457  64.834  -1.629   0.019   0.141   0.840
E R  4Y61A                103    0.563 128.950  -1.733   0.019   0.141   0.840
E G  4Y61A                104    0.377  29.694  -0.761   0.019   0.141   0.840
E F  4Y61A                105    0.385  77.290  -0.349   0.018   0.088   0.893
B P  4Y61A                106    0.146  20.717  -1.217   0.020   0.205   0.775
E T  4Y61A                107    0.482  66.909   0.456   0.004   0.616   0.381
B I  4Y61A                108    0.128  23.662  -0.758   0.002   0.816   0.182
E D  4Y61A                109    0.390  56.199  -0.472   0.018   0.846   0.136
E M  4Y61A                110    0.350  69.975  -0.610   0.004   0.616   0.381
E G  4Y61A                111    0.522  41.097  -0.229   0.004   0.197   0.799
E P  4Y61A                112    0.321  45.592  -1.789   0.005   0.045   0.951
E Q  4Y61A                113    0.567 101.338  -0.409   0.005   0.262   0.733
E L  4Y61A                114    0.487  89.243  -0.959   0.004   0.616   0.381
B K  4Y61A                115    0.187  38.404   1.061   0.002   0.816   0.182
E V  4Y61A                116    0.411  63.201   0.949   0.001   0.959   0.040
B V  4Y61A                117    0.069  10.528   0.318   0.001   0.959   0.040
E E  4Y61A                118    0.446  77.864   1.174   0.001   0.900   0.099
E R  4Y61A                119    0.437 100.096   0.406   0.005   0.336   0.660
E T  4Y61A                120    0.693  96.175  -0.487   0.005   0.045   0.951
E R  4Y61A                121    0.485 111.179   1.149   0.004   0.085   0.910
E T  4Y61A                122    0.542  75.134   1.433   0.004   0.616   0.381
B A  4Y61A                123    0.037   4.088   0.157   0.001   0.959   0.040
E T  4Y61A                124    0.323  44.772   1.899   0.001   0.959   0.040
B M  4Y61A                125    0.034   6.843   0.482   0.001   0.959   0.040
E L  4Y61A                126    0.365  66.777   1.820   0.001   0.959   0.040
B C  4Y61A                127    0.019   2.682   1.099   0.001   0.959   0.040
E A  4Y61A                128    0.421  46.350   1.563   0.001   0.900   0.099
B A  4Y61A                129    0.050   5.543  -0.930   0.001   0.900   0.099
E S  4Y61A                130    0.557  65.316   0.321   0.004   0.616   0.381
E G  4Y61A                131    0.293  23.067  -1.551   0.004   0.197   0.799
E N  4Y61A                132    0.477  69.847  -0.091   0.004   0.138   0.858
B P  4Y61A                133    0.224  31.857  -0.367   0.004   0.138   0.858
E D  4Y61A                134    0.508  73.203   1.061   0.004   0.138   0.858
B P  4Y61A                135    0.077  10.884  -0.255   0.004   0.420   0.576
E E  4Y61A                136    0.416  72.728   1.216   0.001   0.900   0.099
B I  4Y61A                137    0.038   7.030   0.212   0.001   0.959   0.040
E T  4Y61A                138    0.267  36.991   1.802   0.001   0.959   0.040
B W  4Y61A                139    0.034   8.273   0.220   0.001   0.959   0.040
B F  4Y61A                140    0.230  46.201   1.120   0.001   0.959   0.040
B K  4Y61A                141    0.203  41.778   0.891   0.002   0.816   0.182
E D  4Y61A                142    0.624  89.976  -0.654   0.018   0.019   0.964
E F  4Y61A                143    0.687 137.901  -2.006   0.018   0.019   0.964
E L  4Y61A                144    0.497  90.982  -0.139   0.003   0.718   0.279
E P  4Y61A                145    0.482  68.467   0.122   0.002   0.816   0.182
B V  4Y61A                146    0.106  16.261  -0.516   0.023   0.655   0.322
E D  4Y61A                147    0.440  63.375  -0.013   0.021   0.451   0.528
E T  4Y61A                148    0.415  57.533  -1.568   0.021   0.279   0.699
E S  4Y61A                149    0.507  59.385  -1.988   0.018   0.088   0.893
E N  4Y61A                150    0.532  77.943  -2.441   0.018   0.088   0.893
E N  4Y61A                151    0.544  79.656  -1.363   0.020   0.205   0.775
E N  4Y61A                152    0.468  68.442  -1.475   0.020   0.205   0.775
E G  4Y61A                153    0.310  24.366  -1.768   0.021   0.279   0.699
E R  4Y61A                154    0.288  65.975  -0.879   0.023   0.655   0.322
B I  4Y61A                155    0.188  34.688  -1.076   0.021   0.756   0.223
E K  4Y61A                156    0.358  73.723  -0.621   0.021   0.756   0.223
E Q  4Y61A                157    0.340  60.813  -1.252   0.023   0.655   0.322
E L  4Y61A                158    0.347  63.554  -1.210   0.021   0.451   0.528
E R  4Y61A                159    0.530 121.256  -1.552   0.021   0.279   0.699
E S  4Y61A                160    0.562  65.831  -1.699   0.021   0.279   0.699
E E  4Y61A                161    0.377  65.792  -1.517   0.022   0.359   0.619
E S  4Y61A                162    0.458  53.701  -1.409   0.021   0.451   0.528
B I  4Y61A                163    0.282  52.077  -0.996   0.003   0.718   0.279
E G  4Y61A                164    0.357  28.072  -1.404   0.021   0.756   0.223
E G  4Y61A                165    0.343  26.963  -1.268   0.002   0.816   0.182
E T  4Y61A                166    0.412  57.089  -1.343   0.003   0.718   0.279
E P  4Y61A                167    0.386  54.759  -1.309   0.005   0.336   0.660
E I  4Y61A                168    0.504  93.277  -1.652   0.005   0.262   0.733
E R  4Y61A                169    0.514 117.775  -0.511   0.005   0.262   0.733
B G  4Y61A                170    0.098   7.681  -0.575   0.004   0.514   0.481
B A  4Y61A                171    0.211  23.296   0.707   0.002   0.816   0.182
B L  4Y61A                172    0.020   3.699   0.983   0.001   0.900   0.099
E Q  4Y61A                173    0.234  41.721   1.574   0.001   0.959   0.040
B I  4Y61A                174    0.025   4.662   0.596   0.001   0.900   0.099
E E  4Y61A                175    0.416  72.658   1.444   0.002   0.816   0.182
E Q  4Y61A                176    0.524  93.569   0.524   0.004   0.514   0.481
B S  4Y61A                177    0.076   8.907  -0.062   0.005   0.262   0.733
E E  4Y61A                178    0.491  85.813   1.015   0.005   0.262   0.733
E E  4Y61A                179    0.497  86.826   0.336   0.004   0.197   0.799
E S  4Y61A                180    0.619  72.535  -0.374   0.004   0.085   0.910
B D  4Y61A                181    0.195  28.157  -1.049   0.004   0.085   0.910
E Q  4Y61A                182    0.468  83.549  -0.393   0.004   0.197   0.799
B G  4Y61A                183    0.127  10.026  -1.196   0.003   0.718   0.279
E K  4Y61A                184    0.414  85.078   1.794   0.001   0.900   0.099
B Y  4Y61A                185    0.041   8.698   0.681   0.001   0.959   0.040
B E  4Y61A                186    0.232  40.600   2.004   0.001   0.959   0.040
B C  4Y61A                187    0.013   1.769   1.715   0.001   0.959   0.040
E V  4Y61A                188    0.216  33.138   2.245   0.001   0.959   0.040
B A  4Y61A                189    0.026   2.887   0.306   0.001   0.959   0.040
E T  4Y61A                190    0.354  49.141   0.706   0.001   0.900   0.099
B N  4Y61A                191    0.076  11.068  -0.879   0.004   0.616   0.381
E S  4Y61A                192    0.627  73.438   0.067   0.004   0.085   0.910
E A  4Y61A                193    0.425  46.846  -0.872   0.004   0.138   0.858
E G  4Y61A                194    0.377  29.686  -1.381   0.004   0.138   0.858
E T  4Y61A                195    0.597  82.790  -0.874   0.004   0.138   0.858
E R  4Y61A                196    0.521 119.309  -0.939   0.005   0.336   0.660
E Y  4Y61A                197    0.370  79.090  -0.538   0.004   0.514   0.481
E S  4Y61A                198    0.415  48.638  -0.637   0.003   0.718   0.279
E A  4Y61A                199    0.284  31.330   0.265   0.002   0.816   0.182
E P  4Y61A                200    0.385  54.646   0.811   0.001   0.900   0.099
B A  4Y61A                201    0.033   3.659   0.032   0.001   0.959   0.040
E N  4Y61A                202    0.278  40.714   1.674   0.001   0.959   0.040
B L  4Y61A                203    0.023   4.120   0.917   0.001   0.959   0.040
B Y  4Y61A                204    0.194  41.479   1.335   0.001   0.959   0.040
B V  4Y61A                205    0.020   3.120   0.963   0.001   0.959   0.040
E R  4Y61A                206    0.344  78.707   1.218   0.001   0.900   0.099
B E  4Y61A                207    0.224  39.133  -0.185   0.003   0.718   0.279
E L  4Y61A                208    0.296  54.161   0.213   0.021   0.279   0.699
E R  4Y61A                209    0.458 104.951  -1.021   0.019   0.141   0.840
E E  4Y61A                210    0.515  90.058  -0.792   0.021   0.279   0.699
B V  4Y61A                211    0.300  46.033  -1.367   0.021   0.279   0.699
E R  4Y61A                212    0.516 118.141  -1.039   0.020   0.205   0.775
E R  4Y61A                213    0.577 132.247  -1.419   0.020   0.205   0.775
E V  4Y61A                214    0.387  59.420  -0.614   0.019   0.141   0.840
E P  4Y61A                215    0.324  45.933  -0.559   0.018   0.047   0.935
B P  4Y61A                216    0.202  28.678  -1.270   0.021   0.279   0.699
E R  4Y61A                217    0.350  80.173   0.105   0.023   0.655   0.322
B F  4Y61A                218    0.115  23.020  -1.927   0.018   0.846   0.136
B S  4Y61A                219    0.276  32.382  -0.697   0.021   0.756   0.223
E I  4Y61A                220    0.381  70.393  -0.719   0.022   0.552   0.426
E P  4Y61A                221    0.438  62.095  -0.255   0.004   0.197   0.799
B P  4Y61A                222    0.291  41.222  -1.689   0.018   0.088   0.893
E T  4Y61A                223    0.570  79.114  -0.425   0.021   0.279   0.699
E N  4Y61A                224    0.526  77.036  -0.611   0.004   0.514   0.481
B H  4Y61A                225    0.195  35.398   0.750   0.021   0.756   0.223
E E  4Y61A                226    0.443  77.322   0.498   0.011   0.918   0.071
B I  4Y61A                227    0.070  12.932  -0.553   0.002   0.816   0.182
E M  4Y61A                228    0.429  85.843   0.909   0.003   0.718   0.279
E P  4Y61A                229    0.479  67.984   0.246   0.005   0.336   0.660
E G  4Y61A                230    0.639  50.297  -0.998   0.005   0.045   0.951
E G  4Y61A                231    0.457  35.974   0.367   0.004   0.138   0.858
E S  4Y61A                232    0.533  62.479   1.191   0.003   0.718   0.279
B V  4Y61A                233    0.039   6.025  -0.085   0.001   0.900   0.099
E N  4Y61A                234    0.383  56.013   1.983   0.001   0.959   0.040
B I  4Y61A                235    0.046   8.510   0.608   0.001   0.959   0.040
E T  4Y61A                236    0.356  49.405   1.655   0.001   0.959   0.040
B C  4Y61A                237    0.024   3.356   0.903   0.001   0.959   0.040
E V  4Y61A                238    0.383  58.944   1.675   0.001   0.959   0.040
B A  4Y61A                239    0.049   5.433  -0.412   0.001   0.959   0.040
E V  4Y61A                240    0.484  74.360   0.163   0.001   0.900   0.099
B G  4Y61A                241    0.158  12.458  -1.738   0.004   0.514   0.481
E S  4Y61A                242    0.459  53.748   0.323   0.004   0.197   0.799
B P  4Y61A                243    0.168  23.825  -0.619   0.004   0.138   0.858
E M  4Y61A                244    0.494  98.749   0.848   0.004   0.138   0.858
B P  4Y61A                245    0.054   7.719  -0.102   0.004   0.514   0.481
E Y  4Y61A                246    0.359  76.804   1.164   0.001   0.900   0.099
B V  4Y61A                247    0.040   6.194   0.225   0.001   0.959   0.040
E K  4Y61A                248    0.263  54.058   1.858   0.001   0.959   0.040
B W  4Y61A                249    0.031   7.359   0.413   0.001   0.959   0.040
E M  4Y61A                250    0.250  50.005   1.270   0.001   0.959   0.040
B L  4Y61A                251    0.218  39.879   0.997   0.002   0.816   0.182
E G  4Y61A                252    0.556  43.726  -0.130   0.005   0.045   0.951
E A  4Y61A                253    0.719  79.212  -1.298   0.005   0.015   0.979
E E  4Y61A                254    0.573 100.068   0.472   0.004   0.616   0.381
E D  4Y61A                255    0.528  76.070   1.012   0.003   0.718   0.279
B L  4Y61A                256    0.058  10.547  -0.009   0.022   0.552   0.426
E T  4Y61A                257    0.457  63.344   1.225   0.005   0.262   0.733
E P  4Y61A                258    0.618  87.680  -0.771   0.004   0.085   0.910
E E  4Y61A                259    0.697 121.748  -1.442   0.005   0.015   0.979
E D  4Y61A                260    0.571  82.267  -1.859   0.004   0.085   0.910
E D  4Y61A                261    0.471  67.900  -0.460   0.004   0.514   0.481
B M  4Y61A                262    0.258  51.586  -0.153   0.002   0.816   0.182
E P  4Y61A                263    0.449  63.642   0.315   0.002   0.816   0.182
B I  4Y61A                264    0.188  34.817  -1.430   0.003   0.718   0.279
B G  4Y61A                265    0.239  18.770  -1.318   0.004   0.514   0.481
E R  4Y61A                266    0.325  74.310  -0.670   0.004   0.514   0.481
B N  4Y61A                267    0.057   8.345  -1.184   0.002   0.816   0.182
B V  4Y61A                268    0.172  26.436   0.359   0.001   0.959   0.040
B L  4Y61A                269    0.031   5.639   0.336   0.001   0.959   0.040
E E  4Y61A                270    0.369  64.447   1.305   0.001   0.900   0.099
E L  4Y61A                271    0.358  65.513  -0.431   0.002   0.816   0.182
B N  4Y61A                272    0.214  31.271   0.166   0.004   0.420   0.576
E D  4Y61A                273    0.502  72.410  -0.102   0.005   0.336   0.660
E V  4Y61A                274    0.400  61.557   0.029   0.005   0.262   0.733
E R  4Y61A                275    0.593 135.843  -0.318   0.004   0.197   0.799
B Q  4Y61A                276    0.216  38.649  -1.154   0.004   0.197   0.799
E S  4Y61A                277    0.430  50.384  -0.466   0.004   0.197   0.799
B A  4Y61A                278    0.103  11.406  -2.162   0.003   0.718   0.279
E N  4Y61A                279    0.388  56.862   1.812   0.001   0.900   0.099
B Y  4Y61A                280    0.041   8.698   0.652   0.001   0.959   0.040
E T  4Y61A                281    0.255  35.355   2.027   0.001   0.959   0.040
B C  4Y61A                282    0.015   2.148   1.549   0.001   0.959   0.040
E V  4Y61A                283    0.235  36.058   2.151   0.001   0.959   0.040
B A  4Y61A                284    0.031   3.405  -0.390   0.001   0.959   0.040
E M  4Y61A                285    0.370  74.117   0.767   0.001   0.959   0.040
B S  4Y61A                286    0.082   9.657  -0.119   0.004   0.616   0.381
E T  4Y61A                287    0.621  86.147   0.021   0.004   0.138   0.858
E L  4Y61A                288    0.403  73.844  -0.734   0.005   0.262   0.733
E G  4Y61A                289    0.397  31.252  -1.351   0.004   0.514   0.481
E V  4Y61A                290    0.511  78.587  -0.279   0.003   0.718   0.279
B I  4Y61A                291    0.263  48.618  -0.428   0.001   0.900   0.099
E E  4Y61A                292    0.493  86.162   0.405   0.001   0.900   0.099
B A  4Y61A                293    0.148  16.343   0.593   0.001   0.900   0.099
E I  4Y61A                294    0.344  63.732   0.766   0.001   0.959   0.040
B A  4Y61A                295    0.028   3.097   0.286   0.001   0.959   0.040
E Q  4Y61A                296    0.289  51.651   1.896   0.001   0.959   0.040
B I  4Y61A                297    0.017   3.201   1.394   0.001   0.959   0.040
E T  4Y61A                298    0.257  35.674   1.848   0.001   0.959   0.040
B V  4Y61A                299    0.045   6.870   0.146   0.001   0.900   0.099
E K  4Y61A                300    0.402  82.712   0.928   0.002   0.816   0.182
E A  4Y61A                301    0.350  38.537  -0.358   0.004   0.514   0.481
E L  4Y61A                302    0.339  62.034   0.488   0.004   0.138   0.858
B P  4Y61A                303    0.225  31.899  -1.209   0.005   0.045   0.951
E K  4Y61A                304    0.622 128.028  -0.286   0.005   0.045   0.951
B P  4Y61A                305    0.204  28.905   0.034   0.005   0.015   0.979
B P  4Y61A                306    0.233  33.020  -1.031   0.018   0.019   0.964
E G  4Y61A                307    0.572  45.032  -1.353   0.018   0.047   0.935
E T  4Y61A                308    0.585  81.126   0.328   0.018   0.088   0.893
B P  4Y61A                309    0.139  19.781  -0.575   0.005   0.262   0.733
E V  4Y61A                310    0.554  85.165   0.457   0.002   0.816   0.182
B V  4Y61A                311    0.264  40.638  -0.641   0.001   0.900   0.099
E T  4Y61A                312    0.374  51.846   0.169   0.001   0.900   0.099
E E  4Y61A                313    0.538  93.901  -0.461   0.002   0.816   0.182
E S  4Y61A                314    0.344  40.340  -0.099   0.004   0.616   0.381
E T  4Y61A                315    0.602  83.484  -0.728   0.004   0.197   0.799
E A  4Y61A                316    0.618  68.082  -0.668   0.005   0.045   0.951
E T  4Y61A                317    0.457  63.372   0.330   0.005   0.336   0.660
E S  4Y61A                318    0.265  31.070   0.697   0.001   0.900   0.099
B I  4Y61A                319    0.036   6.678  -0.048   0.001   0.959   0.040
E T  4Y61A                320    0.311  43.150   1.551   0.001   0.959   0.040
B L  4Y61A                321    0.038   7.013   0.953   0.001   0.959   0.040
E T  4Y61A                322    0.264  36.561   0.992   0.001   0.959   0.040
B W  4Y61A                323    0.083  19.841   0.121   0.001   0.959   0.040
E D  4Y61A                324    0.419  60.335   0.564   0.002   0.816   0.182
E S  4Y61A                325    0.415  48.591  -0.501   0.005   0.262   0.733
E G  4Y61A                326    0.638  50.234  -2.148   0.005   0.015   0.979
E N  4Y61A                327    0.533  78.075  -1.068   0.003   0.003   0.994
E P  4Y61A                328    0.452  64.082  -2.100   0.005   0.015   0.979
E E  4Y61A                329    0.536  93.604  -0.567   0.005   0.045   0.951
B P  4Y61A                330    0.100  14.190  -1.670   0.005   0.262   0.733
E V  4Y61A                331    0.291  44.681   0.701   0.002   0.816   0.182
B S  4Y61A                332    0.083   9.739   0.262   0.001   0.959   0.040
B Y  4Y61A                333    0.193  41.223   1.139   0.001   0.959   0.040
B Y  4Y61A                334    0.032   6.796   0.544   0.001   0.959   0.040
B I  4Y61A                335    0.192  35.612   1.179   0.001   0.959   0.040
B I  4Y61A                336    0.110  20.350   0.879   0.001   0.959   0.040
E Q  4Y61A                337    0.378  67.457   0.034   0.003   0.718   0.279
E H  4Y61A                338    0.383  69.613  -0.578   0.022   0.359   0.619
E K  4Y61A                339    0.452  92.935   0.064   0.002   0.816   0.182
E P  4Y61A                340    0.508  72.085  -0.463   0.003   0.718   0.279
B K  4Y61A                341    0.238  49.039  -0.786   0.005   0.262   0.733
E N  4Y61A                342    0.497  72.775  -0.738   0.004   0.085   0.910
E S  4Y61A                343    0.645  75.641  -1.795   0.005   0.045   0.951
E E  4Y61A                344    0.671 117.311  -2.478   0.018   0.047   0.935
E E  4Y61A                345    0.534  93.307  -0.913   0.004   0.138   0.858
E P  4Y61A                346    0.377  53.440  -0.326   0.004   0.616   0.381
B Y  4Y61A                347    0.202  43.125   0.385   0.001   0.900   0.099
E K  4Y61A                348    0.369  75.883  -0.330   0.001   0.959   0.040
E E  4Y61A                349    0.302  52.707  -0.411   0.001   0.959   0.040
E I  4Y61A                350    0.342  63.270  -0.747   0.001   0.959   0.040
E D  4Y61A                351    0.335  48.230  -1.528   0.001   0.900   0.099
E G  4Y61A                352    0.505  39.767  -1.144   0.004   0.514   0.481
E I  4Y61A                353    0.393  72.686  -1.572   0.004   0.085   0.910
E A  4Y61A                354    0.714  78.661  -0.903   0.005   0.045   0.951
E T  4Y61A                355    0.532  73.830  -0.879   0.004   0.197   0.799
B T  4Y61A                356    0.124  17.227  -0.679   0.004   0.616   0.381
E R  4Y61A                357    0.287  65.838   0.913   0.001   0.900   0.099
B Y  4Y61A                358    0.032   6.817   0.439   0.001   0.959   0.040
E S  4Y61A                359    0.264  30.906   1.890   0.001   0.959   0.040
B V  4Y61A                360    0.027   4.104   0.468   0.001   0.959   0.040
E A  4Y61A                361    0.464  51.144   1.384   0.002   0.816   0.182
E G  4Y61A                362    0.502  39.484   0.248   0.004   0.616   0.381
B L  4Y61A                363    0.083  15.252  -0.010   0.005   0.262   0.733
E S  4Y61A                364    0.432  50.572   0.988   0.005   0.262   0.733
E P  4Y61A                365    0.551  78.229   0.181   0.005   0.262   0.733
E Y  4Y61A                366    0.579 123.690  -0.668   0.004   0.085   0.910
B S  4Y61A                367    0.227  26.616  -0.426   0.004   0.138   0.858
E D  4Y61A                368    0.442  63.620   0.170   0.004   0.514   0.481
B Y  4Y61A                369    0.094  20.024  -0.743   0.001   0.900   0.099
E E  4Y61A                370    0.329  57.546   2.083   0.001   0.959   0.040
B F  4Y61A                371    0.026   5.178   1.058   0.001   0.959   0.040
B R  4Y61A                372    0.162  37.121   2.196   0.001   0.959   0.040
B V  4Y61A                373    0.011   1.768   1.920   0.001   0.959   0.040
B V  4Y61A                374    0.173  26.605   2.028   0.001   0.959   0.040
B A  4Y61A                375    0.020   2.204   0.528   0.001   0.959   0.040
B V  4Y61A                376    0.234  35.966   0.746   0.001   0.959   0.040
B N  4Y61A                377    0.063   9.267  -0.351   0.004   0.616   0.381
E N  4Y61A                378    0.575  84.195   0.104   0.018   0.088   0.893
E I  4Y61A                379    0.361  66.785  -0.420   0.004   0.197   0.799
B G  4Y61A                380    0.237  18.676  -2.324   0.004   0.420   0.576
E R  4Y61A                381    0.509 116.584  -0.504   0.005   0.336   0.660
B G  4Y61A                382    0.216  16.976  -1.524   0.005   0.262   0.733
E P  4Y61A                383    0.590  83.707   0.201   0.005   0.336   0.660
E A  4Y61A                384    0.305  33.578  -0.507   0.005   0.262   0.733
E S  4Y61A                385    0.331  38.781  -1.256   0.005   0.336   0.660
B E  4Y61A                386    0.168  29.367  -0.595   0.004   0.616   0.381
E P  4Y61A                387    0.362  51.410   1.289   0.001   0.900   0.099
B V  4Y61A                388    0.033   5.011   0.029   0.001   0.959   0.040
E L  4Y61A                389    0.325  59.599   1.669   0.001   0.959   0.040
B T  4Y61A                390    0.038   5.243  -0.234   0.001   0.900   0.099
E Q  4Y61A                391    0.436  77.816   1.483   0.018   0.846   0.136
E K  4Y61A                392    0.330  67.840   0.577   0.004   0.514   0.481
E H  4Y61A                393    0.435  79.054   0.745   0.004   0.197   0.799
E H  4Y61A                394    0.533  96.989   0.052   0.005   0.045   0.951
E H  4Y61A                395    0.703 127.821  -0.211   0.005   0.045   0.951
B H  4Y61A                396    0.150  27.358  -0.146   0.005   0.045   0.951
E H  4Y61A                397    0.544  99.026  -0.349   0.018   0.019   0.964
E H  4Y61A                398    0.853 155.161  -1.941   0.003   0.003   0.994

Explain the output.  Go back.
```

---
